# New Insights into the Non-orthodox Two Component Rcs Phosphorelay System

**DOI:** 10.3389/fmicb.2017.02014

**Published:** 2017-10-17

**Authors:** Xiao-Peng Guo, Yi-Cheng Sun

**Affiliations:** MOH Key Laboratory of Systems Biology of Pathogens, Institute of Pathogen Biology, Chinese Academy of Medical Sciences and Peking Union Medical College, Beijing, China

**Keywords:** Rcs phosphorelay system, envelope stress, acetylation, auxiliary regulators, molecular evolution

## Abstract

The Rcs phosphorelay system, a non-orthodox two-component regulatory system, integrates environmental signals, regulates gene expression, and alters the physiological behavior of members of the Enterobacteriaceae family of Gram-negative bacteria. Recent studies of Rcs system focused on protein interactions, functions, and the evolution of Rcs system components and its auxiliary regulatory proteins. Herein we review the latest advances on the Rcs system proteins, and discuss the roles that the Rcs system plays in the environmental adaptation of various Enterobacteriaceae species.

## Introduction

**FIGURE 1:**
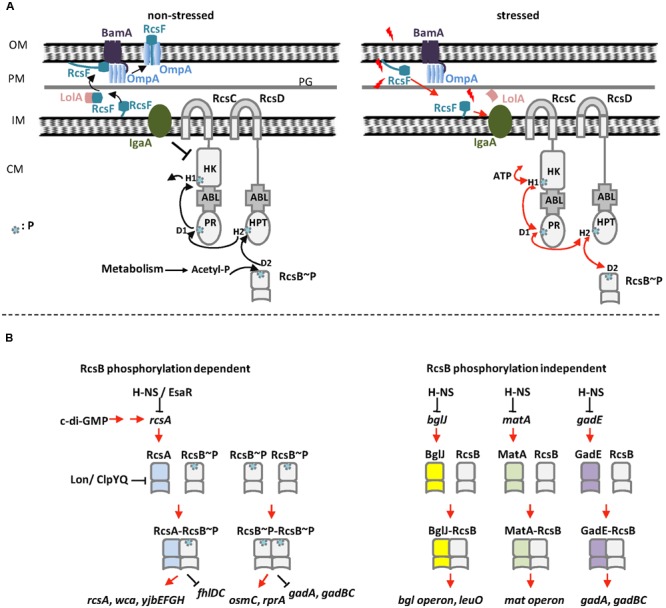
The Rcs phosphorelay system in *E. coli*. **(A)** Regulation of RcsB phosphorylation in *E. coli*. Left: in the absence of stress, RcsF is funneled by BamA and sequestered by OmpA as a surface-exposed protein. YrfF (IgaA in *S. enterica* serovar Typhimurium) inhibits the kinase activity of RcsC, resulting in dephosphorylation of RcsB. In this situation, AcP might act as a phosphoryl group donor to keep a low level of RcsB phosphorylation. Right: in the presence of stress, RcsF does not interact with BamA and OmpA but interacts with IgaA that release the inhibition of YrfF (IgaA in *S. enterica* serovar Typhimurium). RcsC autophosphorylates at the H1 position of its HK domain in an ATP-dependent manner. The phosphoryl group is then transferred to D1 on the PR domain of RcsC, then to H2 on the HPt domain of RcsD and finally to RcsB. **(B)** The homo- and heterodimers of RcsB in *E. coli*. Left: RcsB forms homodimer or heterodimer with RcsA to regulate target genes in a phosphorylation dependent manner. Right: RcsB forms heterodimer with BglJ, GadE, or MatA to regulate target genes in a phosphorylation independent manner. OM, outer membrane; PM, periplasm; IM, inner membrane; CM, cytoplasm; PG, peptidoglycan; P, phosphoryl group.

The Regulator of Capsule Synthesis phosphorelay system was originally identified as a positive regulator of colanic acid capsule in *Escherichia coli* (Gottesman et al., 1985). Colanic acid capsule is composed of glucose, galactose, and glucuronic acid. Overproduction of colanic acid results in a distinctive ‘mucoid’ colony phenotype (Gottesman et al., 1985). Subsequently studies have shown that Rcs phosphorelay system is a non-orthodox two-component signal transduction system (TCS) present in members of the Enterobacteriaceae family of Gram-negative bacteria (Gottesman et al., 1985; Allen et al., 1987; Brill et al., 1988; Majdalani and Gottesman, 2005; Huang et al., 2006). The Rcs system is composed of three core proteins, the transmembrane sensor kinase RcsC, the transmembrane protein RcsD, and the response regulator RcsB (Majdalani and Gottesman, 2005). The response regulator RcsB and the membrane-localized hybrid sensor kinase RcsC represent the classical members of bacterial TCS (Stout and Gottesman, 1990), whereas the membrane-bound sensor RcsD lacks kinase activity (Majdalani and Gottesman, 2005; Clarke, 2010). Several accessory components have also been described, such as auxiliary regulators RcsA, BglJ, GadE, MatA (EcpR), DctR, TviA, RflM, and RmpA (**Table 1**; McCallum and Whitfield, 1991; Virlogeux et al., 1996; Winter et al., 2009; Castanié-Cornet et al., 2010; Cheng et al., 2010; Venkatesh et al., 2010; Kühne et al., 2016; Pannen et al., 2016), and lipoprotein RcsF that senses envelope stress (Gervais and Drapeau, 1992; Majdalani et al., 2005; **Figure 1A**). As a non-orthodox TCS, the Rcs system engages in complex multistep phosphorelay signal transduction (**Figure 1**; Takeda et al., 2001; Majdalani and Gottesman, 2005). Upon receiving an extracytoplasmic stimulus, likely via RcsF, the hybrid sensor RcsC autophosphorylates at a conserved histidine residue (His479 in RcsC from *E. coli*) on its histidine kinase (HK) domain in an ATP-dependent manner. The phosphoryl group is then transferred to an aspartate residue (Asp875 in RcsC from *E. coli*) on the phosphoryl receiver (PR) domain of RcsC, and subsequently to a histidine residue (His842 in RcsD from *E. coli*) on the unique C-terminal histidine-containing phosphotransmitter (HPt) domain of RcsD. Finally, the phosphoryl group is transmitted to an aspartate residue (Asp56 in RcsB from *E. coli*) on the PR domain of RcsB (Chen et al., 2001; Takeda et al., 2001; Clarke et al., 2002; Majdalani and Gottesman, 2005). In addition, in the absence of extracytoplasmic stress or in response to certain metabolic stress, RcsB can also be phosphorylated by the central metabolite acetyl phosphate (AcP), an intracellular low-molecular-weight phosphoryl group donor (Fredericks et al., 2006; Hu et al., 2013).

**Table 1:** Homodimer and heterodimers of RcsB.

Dimer	Species	Function(s) of the dimer	Reference
RcsB–RcsB	*E. coli* and others	Activation of *rprA* which positively regulates RpoS in *E. coli*, repression of glutamate decarboxylase *gadA* in *E. coli* and etc.	Ferrières et al., 2009;Castanié-Cornet et al., 2010

RcsA–RcsB	*E. coli* and others	Activation of *cps* operon involved in capsular polysaccharide biosynthesis in *E. coli*, activation of *yjbEFGH* involved in EPS synthesis in *E. coli* and etc.	Stout et al., 1991;Majdalani and Gottesman, 2005;Ferrières et al., 2007

BglJ–RcsB	*E. coli*	Activation of *bgl* loci involved in aryl-β, D-glucoside synthesis, activation of transcription factor *leuO* and etc.	Venkatesh et al., 2010;Stratmann et al., 2012

GadE–RcsB	*E. coli*	Activation of *gad* genes involved in acid resistance	Castanié-Cornet et al., 2010

MatA–RcsB	*E. coli*	Activation of *mat* operon involved in Mat fimbria synthesis	Pannen et al., 2016

DctR–RcsB	*E. coli*	Protection against organic acids while none of direct target genes were identified	Pannen et al., 2016

RmpA–RcsB	*K. pneumoniae*	Activation of *cps* operon involved in capsular polysaccharide biosynthesis	Cheng et al., 2010

RflM–RcsB	*S. enterica*	Repression of *flhDC* which is involved in flagellar synthesis	Kühne et al., 2016

TviA–RcsB	*S. typhi* and *S. enterica*	Activation of *viaA* and *viaB* involved in Vi antigen synthesis	Virlogeux et al., 1996;Winter et al., 2009

**Highlights**

**Regulator of Capsule Synthesis phosphorelay system:** a non-orthodox two-component signal transduction (TCS) system with multistep cascade, His–Asp–His–Asp, by three core proteins RcsC, RcsD, and RcsB.

**Envelope stress:** stress by alteration of bacterial envelope that is a complex extracytoplasmic compartment.

**Lysine acetylation:** a reversible posttranslational modification of proteins and plays a key role in regulating gene expression, with an addition of an acetyl moiety to the 𝜀-amino group of a lysine residue.

**Molecular evolution:** the process of change in the sequence composition of cellular molecules such as DNA, RNA, and proteins across generation.

RcsB can form homodimer or heterodimers with the auxiliary proteins, which then interact with a conserved motif in target genes to modulate their transcription. The involvement of a hybrid sensor kinase, additional functional domains, multiple phosphodonors, several acceptor sites, and a variety of auxiliary proteins results in a high complex signaling system, which likely increases the flexibility of the Rcs system and provides multiple checkpoints to facilitate precise regulation of gene expression. Recent studies on the Rcs system focused on protein interactions, functions, modifications, and on the evolution of the Rcs components. Herein we review the latest advances of the complex Rcs system and discuss the mechanisms by which Rcs system senses environmental changes and regulates gene expression to allow Enterobacteriaceae species to adapt to different environments.

## Inputs Regulating RCS Regulon

The first environmental signal reported to activate the Rcs system was osmotic upshift in *E. coli*, and several other regulatory inputs have been discovered since then (Sledjeski and Gottesman, 1996). The activation or repression of Rcs system, in turn, regulates its target genes to relieve the damages caused by the input signals. For example, lysozyme induces Rcs system, which upregulates the transcription of lysozyme inhibitors in *E. coli* (Callewaert et al., 2009). Similarly, the Rcs system is activated upon oxidative stress in *Salmonella*, which regulates the transcription of *dps* gene that protects the bacterial DNA against damage resulting from host reactive oxygen species (ROS) production during infection (Farizano et al., 2014). In the following section, we will discuss several examples of inputs that regulate the Rcs system.

### Perturbation of the Envelope Leads to Activation of Rcs System

The envelope protects the bacterial cell from toxic substances or environmental stress. Extracellular or intracellular signals may cause perturbation of the cell envelope, which activates the Rcs system (Majdalani et al., 2005). In *E. coli*, these signals include the damage of periplasmic peptidoglycan caused by lysozyme (Callewaert et al., 2009), the transient cell wall-deficient (or L-form) state caused by exposure to β-lactam antibiotics (Hirakawa et al., 2003; Glover et al., 2009), defective lipoprotein sorting by *lolA* mutation (Tao et al., 2012), loss of the periplasm-spanning protein TolA (Vianney et al., 2005; Morgan et al., 2014), and modification of the peptidoglycan in the absence of certain penicillin-binding proteins PBP4, PBP5, PBP7, or AmpH (**Table 2**; Evans et al., 2013). Rcs system is also induced by perturbation of the cell envelope in other bacteria. For example, a change in the concentration of osmoregulated periplasmic glucans (OPGs) in *Dickeya dadantii* (Bontemps-Gallo et al., 2013), the absence of UDP-glucose dehydrogenase in *Edwardsiella tarda* (Lv et al., 2012), alteration in the enterobacterial common antigen (ECA) structure in *Serratia marcescens* (Castelli and Vescovi, 2011), and the outer membrane damage caused by oxidative stress in *Salmonella* can modulate Rcs phosphorelay system (**Table 2**; Farizano et al., 2014). In summary, the perturbation of the envelope caused by these extracellular or intracellular signals can be sensed by Rcs system, which in turn regulates its target genes to allow cells to adapt to the environmental or genetic changes.

**Table 2:** Extracellular or intracellular signals that cause envelope stress and induce Rcs system.

Signal	Description	Species	Function	Reference
Lysozyme	*N*-acetylmuramide glycanohydrolase	*E. coli*	Destruction of the periplasmic peptidoglycan	Callewaert et al., 2009

β-lactam antibiotics	Chemicals repressing penicillin binding protein (PBPs)	*E. coli*	Inhibition of peptidoglycan formation and transition to L-form state	Hirakawa et al., 2003; Glover et al., 2009

*lolA* mutation Ile93-Cys/Phe140Cys	Lipoprotein-specific chaperone *lolA*	*E. coli*	Defect in lipoprotein sorting	Tao et al., 2012

*pbp4, pbp5, pbp7*, or *ampH* deletion	Penicillin-binding protein *pbp4*, etc.	*E. coli*	Modification of the peptidoglycan	Evans et al., 2013

*tolA* deletion	Periplasm spanning protein *tolA*	*E. coli*	Outer membrane perturbation	Morgan et al., 2014

*mdoH* deletion	*mdoH*, glucosyltransferase for the synthesis of periplasmic membrane-derived oligosaccharides	*E. coli*	Defect in membrane-derived oligosaccharides synthesis	Shiba et al., 2012

The concentration of OPGs	OPGs, osmoregulated periplasmic glucans	*D. dadantii*	Sensing the osmolarity of medium	Bontemps-Gallo et al., 2013

*ugd* deletion	UDP-glucose dehydrogenase *ugd*	*E. tarda*	Defect in LPS, a truncated core with no O-antigen attached	Lv et al., 2012

*wecG* mutation	ManNAcA transferases *wecG*	*S. marcescens*	Alteration in the enterobacterial common antigen (ECA) structure	Castelli and Vescovi, 2011

### Detecting Envelope Stress via RcsF

RcsF, a small lipoprotein (14 kDa), is membrane-associated via a lipidated N-terminal membrane-anchored helix at the outer membrane, with a loop and a central four-stranded β-sheet in the periplasmic space (Castanié-Cornet et al., 2006). Acting as a mediator between environmental stimuli and Rcs phosphorelay system, RcsF activates Rcs system signaling in response to different types of envelope stress (Shiba et al., 2012; Laloux and Collet, 2017). The mechanism by which envelope stress induces the Rcs system via RcsF has been recently investigated by Silhavy’s lab and Collet’s lab (Cho et al., 2014; Konovalova et al., 2014). Both of them found that Rcs system interacts with BamA and three β-barrels (OmpA, OmpF, and OmpC) to detect envelope stress. Cho et al. (2014) proposed that the interaction between RcsF and BamA plays a major role in RcsF sensing (**Figure 1A**). In the absence of envelope stress, RcsF is trapped by the outer membrane β-barrel assembly machinery (Bam) and funneled to β-barrels (Cho et al., 2014; Konovalova et al., 2014). In this situation, RcsF cannot interact with YrfF (IgaA in *Salmonella enterica* serovar Typhimurium) to activate Rcs system (**Figure 1A**). In the presence of envelope stress, the interaction between RcsF and BamA is impaired and therefore RcsF cannot be funneled to OmpA/C/F. This allows RcsF to be exposed to the periplasm and free to activate Rcs system (**Figure 1A**). This model proposed that RcsF senses envelope stress by monitoring the Bam complex (Cho et al., 2014). Konovalova et al. (2014, 2016) proposed another model to explain how RcsF senses the lipopolysaccharide (LPS) defects. In their model, RcsF forms a complex with β-barrels and uses its positively charged, surface-exposed N-terminal domain to directly monitor lateral interactions between LPS molecules, which in turn regulate Rcs system (Konovalova et al., 2014, 2016). These two models might represent two different ways for RcsF to sense different types of stress (as reviewed in Laloux and Collet, 2017). Their findings elucidate the mechanism by which RcsF senses and transmits signals as a sentinel in the transduction signal pathway and are important to dissect the regulatory mechanism of Rcs system.

The functional N-terminal domain of RcsF contains five basic amino acid residues and six prolines (Umekawa et al., 2013). The positively charged residues are important for RcsF to sense alterations in LPS (Konovalova et al., 2016). The prolines might also be important for RcsF to sense environmental signals but their role has not been investigated. RcsF has two non-consecutive disulfide bridges, Cys74–Cys118 and Cys109–Cys124 in the *E. coli* protein, whose formation is catalyzed by the periplasmic disulfide isomerase DsbC (Leverrier et al., 2011; Rogov et al., 2011). Both of the disulfide bridges Cys74–Cys118 and Cys109–Cys124 are important for the activity of RcsF (Rogov et al., 2011). However, whether the formation of disulfide bridges is involved in sensing the environmental signal also needs to be investigated. Future work should continue to reveal the detailed mechanism of RcsF sensing at the molecular level.

### Factors Regulating the Expression and Stability of Rcs System Proteins

Rcs system can be regulated by the expression level or stability of Rcs system proteins (Majdalani and Gottesman, 2005). For example, expression of *rcsA* is autoregulated by the RcsA–RcsB heterodimer in *E. coli, S. enterica*, and *Klebsiella pneumoniae* (Wang and Harshey, 2009). *rcsB* is transcribed from two promoters, P*rcsDB* and P*rcsB* in *S. enterica* serovar Typhimurium (Wang and Harshey, 2009; Pescaretti Mde et al., 2010). RcsB represses *rcsD* gene expression by binding directly to the P*rcsDB* promoter, negatively autoregulating *rcsB* in *S. enterica* serovar Typhimurium (Pescaretti Mde et al., 2010). Furthermore, the *rcsA* gene is indirectly upregulated by c-di-GMP in *E. coli* (Sledjeski and Gottesman, 1995; Mendez-Ortiz et al., 2006) and negatively regulated by the quorum-sensing regulator EsaR in *Pantoea stewartii* (Carlier and von Bodman, 2006).

All auxiliary regulators seem to be repressed by the nucleoid-associated global repressor protein H-NS (Sledjeski and Gottesman, 1995; Krin et al., 2010; Venkatesh et al., 2010; Martinez-Santos et al., 2012; Pannen et al., 2016). In addition, transcription of *dctR* is activated by the regulator YdeO in response to extracellular acid stress (Yamanaka et al., 2014). The expression of *matA* in the *mat* (*ecp*) operon encoding fimbrial adhesin is induced at acidic pH, low temperature, or high acetate concentration (Lehti et al., 2013). Finally, RcsA is unstable at 37°C and can be degraded by the ClpYQ and Lon proteases (Allen et al., 1987; Stout et al., 1991; Chang et al., 2016). The regulatory roles of RcsB heterodimers are largely controlled by the availability and regulation of the auxiliary regulators.

## Functions and Interactions of RCS System Components

As mentioned above, Rcs system components include RcsC, RcsD, RcsB, and auxiliary regulators such as RcsA. Compared to other TCS proteins, Rcs system components exhibit high sequence and structural specificity among homologs (Majdalani and Gottesman, 2005; Clarke, 2010). In this section, we summarize the latest advances in our knowledge of the functions and interactions of Rcs system proteins.

### RcsC Performs Dual Functions

RcsC is located at the inner membrane, with most of the protein located in the cytoplasm and a small periplasmic domain in the periplasmic space (Löhr et al., 2005). The atomic structure of the C-terminus of *E. coli* RcsC was determined by NMR, revealing a novel α–β-loop (ABL) domain and a PR domain (Löhr et al., 2005; Rogov et al., 2006). Upon phosphorylation of the RcsC-PR domain, it is recognized by RcsD-HPt and phosphoryl transmission takes place (Löhr et al., 2005). Meanwhile, RcsC-ABL facilitates phosphoryl transfer between RcsC-HK and RcsC-PR, or between RcsC-PR and RcsD-HPt (Rogov et al., 2006). These interactions mediate the signal transduction function of RcsC, while the first identified ABL domain of RcsC revealed a coordination module. Thus, RcsC contributes to the complexity of prokaryotic signaling systems.

RcsC was initially identified as a kinase in the Rcs phosphorelay system. As we know, many kinases are also phosphatases, and subsequent studies showed that RcsC also possesses phosphatase activity (Garcia-Calderon et al., 2005). RcsC functions together with RcsD as a phosphatase to precisely control the low levels of phosphorylated RcsB in the cell in the absence of environmental signal. Residues Ala904, Asp875, and Thr903 are required for the putative phosphatase activity of RcsC-PR (Garcia-Calderon et al., 2005). Recently, Ancona et al. (2015) reported that RcsC also exhibits dual functionality in *Erwinia amylovora*. Mutation of Thr904 (corresponding to Thr903 in *E. coli* RcsC) and Ala905 (corresponding to Ala904 in *E. coli* RcsC) constitutively activates the Rcs system (Ancona et al., 2015). The dual functions of RcsC are essential for its regulatory role to prevent the constitutive activation of the Rcs system. However, the molecular mechanism of its phosphatase activity is not yet clear.

### Interactions between RcsD and Other Rcs System Proteins

RcsD is an inner membrane protein with a substantial periplasmic domain. Unlike RcsC, RcsD does not show any autophosphorylation activity, due to the lack of a canonical active site (Majdalani and Gottesman, 2005). The structure of the HPt domain of *E. coli* RcsD has been determined and shown to be similar to ArcB-HPt (Rogov et al., 2004). RcsD-HPt has a recognition area in close vicinity to the phosphoryl site (His842) that is recognized by two cognate PR domains, RcsB-PR and RcsC-PR. The interaction interfaces of the two domains with RcsD-HPt largely overlap. Meanwhile, Schmoe et al. (2011) describe a complex between RcsD-ABL and RcsB. Earlier Rogov et al. (2006) describe a complex between RcsC-ABL and RcsD. These complexes probably enhance the interaction between membrane-bound proteins and soluble components to increase the rate and efficiency of the phosphorelay signaling cascade (Schmoe et al., 2011).

### Post-translational Modification of RcsB

The C-terminal DNA-binding domain of RcsB, belonging to the FixJ/NarL family, forms a helix-turn-helix (HTH) DNA-binding motif (Pristovsek et al., 2003). The N-terminal domain of RcsB, containing the phosphoacceptor site, belongs to the family of two-component receiver domains (Filippova et al., 2016). This receiver domain participates in RcsB dimer formation and also contributes to dimer formation with other transcription factor partners. In response to environmental stimuli, RcsB is phosphorylated by RcsD, and phosphorylation modulates its own DNA-binding affinity at the promoter region of target genes (Majdalani and Gottesman, 2005; Ancona et al., 2015). The phosphorylation status of RcsB is critical for biofilm development in *S. enterica* serovar Typhimurium (Latasa et al., 2012). Phosphorylated RcsB stimulates the transcription of sRNA *rprA*, and the accumulation of *rprA* leads to repression of the transcriptional regulator CsgD, resulting in inhibition of biofilm development (Latasa et al., 2012). In the absence of environmental signals, RcsC and RcsD might act as a phosphatase to dephosphorylate RcsB to maintain a low level of *rcsB* expression (Clarke, 2010; Hu et al., 2013). In this situation, AcP, the intermediate of the Pta-AckA pathway, might provide the phosphoryl group for RcsB, ensuring a low level of RcsB phosphorylation (Hu et al., 2013). Recently, Filippova et al. (2016) solved the crystal structure of non-phosphorylated receiver domain of RcsB. They found that the RcsB receiver domain can exist as a dimer in solution (Filippova et al., 2016). They suggested the non-phosphorylated and phosphorylated states of RcsB use a similar dimerization interface in the homodimer or heterodimers (Filippova et al., 2016). How phosphorylation affects the DNA binding ability and regulatory roles of RcsB is still not clear.

Another RcsB modification is lysine acetylation. RcsB can be acetylated by AcP and deacetylated by CobB (Thao et al., 2010; Hu et al., 2013). Thao et al. (2010) found that Lys180 of RcsB can be acetylated *in vitro*, while Hu et al. (2013) reported that several lysines in RcsB can be acetylated *in vivo* and acetylation of Lys154 negatively regulates RcsB activity. Castano-Cerezo et al. (2014) reported that acetylation of RcsB prevents DNA binding, activates flagella biosynthesis and motility, and compromises acid stress survival. Their finding indicates that acetylation might play an important role as same as phosphorylation in regulation of the activity of RcsB. Although Rcs system is important for enterobacteria, constitutive activation of the Rcs system could reduce the fitness and virulence (Ancona et al., 2015). Thus phosphorylation and acetylation might act together to tightly control the activity of RcsB, which in turn fine-tunes the function of Rcs system to adapt to the differential environment. The inter-conversion between phosphorylation and acetylation of RcsB remains largely unclear and needs to be investigated.

### Homodimer and Heterodimers of RcsB

RcsB binds DNA either as a homodimer or heterodimer with auxiliary regulators (**Table 1**). The auxiliary proteins cooperate with RcsB to modulate transcription by binding to specific DNA motifs. The motifs binding by different dimers have a similarity in one half that is probably bound by RcsB, whereas the other half varies. The interaction of RcsB with auxiliary proteins might alter the specificity or strengthen the ability of DNA binding (Salscheider et al., 2014).

RcsB homodimer regulates targets once RcsB is phosphorylated. The binding sites are usually located upstream of the -35 region of the RNA polymerase-binding site (Majdalani and Gottesman, 2005). RcsB homodimer positively regulates sRNA *rprA* and the osmoregulated gene *osmC* in *E. coli*, as well as other targets in an RcsB phosphorylation-dependent manner (Majdalani and Gottesman, 2005; Clarke, 2010). RcsA stabilizes RcsA–RcsB complex to the RcsAB box and enhances the regulation activity of RcsB (Majdalani and Gottesman, 2005; Clarke, 2010). It is worth noting that RcsA’s function is dependent of phosphorylation of RcsB, whereas the other auxiliary regulators such as MatA, BglJ, and GadE act independently of phosphorylation of RcsB (Castanié-Cornet et al., 2010; Venkatesh et al., 2010; Pannen et al., 2016). For phosphorylation-independent heterodimers, it is not the level of RcsB phosphorylation in the cytoplasm but rather the presence of different auxiliary regulators that is important for its functions (Pannen et al., 2016). In addition, GadE–RcsB heterodimer activates the transcription of glutamate-dependent (Gad) acid resistant genes *gadA/BC*, but acetylation of RcsB negatively regulates *gadA*/*BC* (Castanié-Cornet et al., 2010; Castano-Cerezo et al., 2014). This indicates the function of phosphorylation-independent heterodimers might be affected by the acetylation of RcsB.

Castanié-Cornet et al. (2010) and Venkatesh et al. (2010) reported that BglJ–RcsB and GadE–RcsB heterodimers are active independent of RcsB phosphorylation. Pannen et al. (2016) found that MatA–RcsB heterodimer is also active independent of RcsB phosphorylation. In addition, they investigated conserved or surface-exposed residues and their roles in the activity of RcsB in *E. coli*. Their results revealed that mutation of residues in the vicinity of the phosphorylation site such as Asp56, Asp11, Thr87, and Lys109 impairs the activity of phosphorylation-dependent dimers such as RcsA–RcsB and RcsB–RcsB. Meanwhile, residue Lys109 also affects the activity of phosphorylation-independent heterodimers such as BglJ–RcsB and MatA–RcsB. One possible explanation is that a phosphorylation-induced structural change releases the DNA-binding domain that is otherwise buried or inhibited by the non-phosphorylated form of the PR domain. For both phosphorylation-dependent and -independent dimers, mutation of surface-exposed residue Ile14 impairs the activity of the dimers, indicating that RcsB and its auxiliary proteins interact via a surface similar to that with which NarL and related proteins interact (Pannen et al., 2016). Their findings suggest that phosphorylation might cause a structure change to stabilize the active form of RcsB–RcsB and RcsA–RcsB, while the heterodimers of BglJ–RcsB and MatA–RcsB are intrinsically active.

The heterodimers formed by the transcriptional regulator RcsB with auxiliary regulatory proteins modulate the DNA binding specificity and expands the regulatory role of Rcs system. In addition, the flexibility of the Rcs system regulatory role is further increased because some of the RcsB heterodimers depend on RcsB phosphorylation but some of them do not depend on RcsB phosphorylation. Multiple auxiliary regulators with varying dependence on the phosphorylation of RcsB endow flexibility to the Rcs system that may allow the bacteria to adapt to diverse environments.

## Targets of the RCS Regulon

Rcs system was first reported to regulate the expression in *E. coli* (Gottesman et al., 1985). Later many Rcs system regulated genes were identified in enterobacteria using transcriptome or proteome analysis (Hinchliffe et al., 2008; Bury-Mone et al., 2009; Mariscotti and Garcia-del Portillo, 2009; Wang et al., 2012). Rcs system predominantly regulates genes involved in the production of important cell surface-associated structures (e.g., flagella, LPS, and fimbriae), exopolysaccharide, and environmental stress-related genes (Fredericks et al., 2006; McMahon et al., 2012; Tao et al., 2012; Farizano et al., 2014; Fang et al., 2015). Rcs phosphorelay system regulates these genes to remodeling the cell surface or metabolism to adapt to the stressed environment. In this section, we discuss the Rcs-regulated target genes involved in biofilm formation, acid resistance, and virulence.

### Role of Rcs System in Biofilm Formation in *Y. pestis*

The plague pathogen *Y. pestis* forms biofilms in the midgut of its flea host to enhance transmission, and cyclic-di-GMP positively regulates biofilm development. In *Y. pestis*, c-di-GMP is synthesized by diguanylate cyclases HmsT and HmsD, and degraded by HmsP (Sun et al., 2011). We showed that the transcription of *hmsT* is directly repressed by RcsB in *Y. pestis* (Sun et al., 2012). RcsB binds to a conserved Rcs-binding motif which overlaps with the transcriptional start site of *hmsT*, leading to the repression of the transcription of *hmsT* (Sun et al., 2012). In addition, we also found that RcsB positively and directly regulates the *hmsCDE* operon (Guo et al., 2015). Furthermore, Fang et al. (2015) found that RcsB also directly represses the transcription of *hmsHFRS*, which is responsible for the biosynthesis and translocation of biofilm matrix exopolysaccharide. In summary, the Rcs system acts as a master repressor of biofilm formation by regulating the production of cyclic-di-GMP and biofilm matrix exopolysaccharide synthesis to facilitate environmental adaptation in *Y. pestis*.

### Other Functions of Rcs System

Hundreds of genes were found to be regulated by the Rcs system using genome-wide approaches in *E. amylovora, E. coli, Y. pestis*, and *S. enterica* (Hinchliffe et al., 2008; Bury-Mone et al., 2009; Mariscotti and Garcia-del Portillo, 2009; Wang et al., 2012). Numerous Rcs-associated phenotypes have been characterized including quorum sensing in *E. coli* (Shimada et al., 2013), cell division in *Proteus mirabilis* (Howery et al., 2015), lipoprotein sorting in *E. coli* (Tao et al., 2012), and biogenesis of outer membrane vesicles in *S. marcescens* (McMahon et al., 2012). Recent advances on the targets of the Rcs system have focused on acid resistance and virulence.

The Gad system is the most efficient acid resistant system in *E. coli* (Foster, 2004; De Biase and Pennacchietti, 2012; Lund et al., 2014). RcsB controls the expression of *gad* genes and is required for acid resistance in *E. coli* (Castanié-Cornet et al., 2007). Castanié-Cornet et al. (2007, 2010) reported that the basal activity of RcsB is necessary and sufficient for GadE dependent regulation of *gad* genes, whereas increased RcsB activity through activation of the Rcs phosphorelay system or through overproduction of the protein leads to general repression of the *gad* genes. This result is consistent with the later findings that the regulatory role of RcsB/GadE is independent of RcsB phosphorylation (Castanié-Cornet et al., 2010), while the regulatory role of RcsB homodimer is dependent of RcsB phosphorylation. Further studies showed that activation of transcription *gadA*, encoding a glutamate decarboxylase, requires binding of RcsB/GadE to a GAD box, while repression of *gadA* transcription is caused by binding of RcsB to an RcsB box which lies in the -10 and -35 regions of *gadA* promoter (Castanié-Cornet et al., 2010). In response to acid stress, RcsB might function together with GadE to precisely regulate the expression of acid resistance-related genes (Castanié-Cornet et al., 2007; Johnson et al., 2011). However, a number of acid resistance-related genes including *safA, slp*, and *gadW* are regulated by RcsB but not GadE (Johnson et al., 2011). Johnson et al. (2011) speculated that RcsB might work with the response regulator EvgA as a heterodimer to regulate acid resistance-related genes.

The Rcs system also contributes to bacterial virulence. LcrF, a master transcriptional regulator of virulence genes in *Yersinia pseudotuberculosis*, is regulated by Rcs system (Li et al., 2015). Rcs system can also modulate the interaction between pathogen and host. For example, RcsB regulates the colonization of *E. coli* on the mouse intestine (Lasaro et al., 2014). In addition, deletion of cytoskeletal element *mreC* induces RcsC, resulted in downregulation of flagella systems and the SPI-1 type 3 secretion system (T3SS) and attenuated virulence in *Salmonella* (Bulmer et al., 2012). In *S. marcescens*, RcsB also represses the pore-forming toxin ShlA that is responsible for early induction of autophagy in host cells (Di Venanzio et al., 2014). Therefore, the Rcs system can affect the interaction between pathogen and host by regulating the assembly of the secretion system, colonization of the host, and early induction of autophagy in host cells.

## Adaptation of the RCS Regulon Genes

Mutation of genes, especially those encoding global regulators such as the Rcs system, can alter cellular signaling circuits, expression of target genes, and physiological behavior, which in turn affect the ability of an organism to adapt to its environment. In the following section, we will discuss the examples of the molecular evolution of Rcs system genes as far as we know.

### RcsA and RcsD in *Y. pseudotuberculosis* and *Y. pestis*

The *rcsA* gene in *Y. pestis* contains a 30 bp internal duplication, and is therefore nonfunctional, whereas *rcsA* in the ancestral *Y. pseudotuberculosis* species is functional and can repress biofilm formation. When *Y. pestis* is supplemented with the functional *Y. pseudotuberculosis* RcsA, biofilm formation is repressed and even abolished in fleas. Therefore, the conversion of *rcsA* to a pseudogene during the evolution of *Y. pestis* represents a case of positive selection (Sun et al., 2008). Furthermore, the loss of *rcsA* in *Y. pestis* following the gain of phospholipase D encoded by *ymt*, together with the loss of two minor genes in the bacterial progenitor, facilitated the transmission of fleas by stimulating c-di-GMP-mediated biofilm formation in the flea foregut (Sun et al., 2014). The evolutionary history of *rcsA* in *Y. pestis* is one example that a few genetic changes such as gene losses can alter the regulation of targets and consequently the infection phenotype, leading to arthropod–borne transmission. Despite an apparent frameshift, RcsD remains functional and acts in concert with RcsB to promote biofilm formation in *Y. pestis*, but it functions in a way that is opposite to RcsD in *Y. pseudotuberculosis* (Sun et al., 2008). The effect of mutating *rcsD* on Rcs system signal transduction and its biological significance requires further experimental investigation.

### RcsB in *E. coli* O157: H7

*Escherichia coli* O157: H7 (O157) is a frequent cause of foodborne disease in humans, and is transmitted to human through food vehicles. To cause infection, O157 needs to adapt to and survive in diverse conditions. Curli fimbriae play an important role in surface attachment, biofilm formation, and confer cell protection from toxic compounds. The production of curli in *E. coli* O157: H7 is intrastrain and interstrain variant (Carter et al., 2012). The curli producing (C+) O157 variants are much more acid sensitive, but grow better in nutrient-limited environment than curli-deficient (C-) variants. Deletion of *rcsB* gene is responsible for the C+ phenotypes in two O157 strains, isolated during the 1993 U.S. hamburger-associated outbreak (Carter et al., 2012). In addition, the C+ O157 variants can also be generated by insertion or single nucleotide change in *rcsB* gene (Chen et al., 2016; Sharma et al., 2017). RcsB affects bacterial survival in growth-restrictive environments through regulation of genes related to biofilm formation and certain metabolic functions. Inactivation of *rcsB* in O157 might enhance the persistence and survival ability in nutrient limited environment, but decrease the resistance to heat shock and acid stress. Thus, mutation of *rcsB* generates a distinct O157 subpopulation that may confer this pathogen with survival advantages in the ever changing environment.

## Conclusion and Future Perspectives

Recent advances on the Rcs system have helped to unravel the interactions, functions, and evolution of Rcs system proteins. Moreover, knowledge on the auxiliary regulatory proteins, regulation of the Rcs regulon, targets, and physiological behavior has considerably extended. Modifications of RcsB, dual functions of RcsC, involvement of multiple auxiliary proteins, and adaptation of Rcs system genes contributed together to increase the complexity and flexibility of the Rcs system that leads to the precise control of its target genes. However, many questions concerning Rcs system structure and function remain unclear. For example, the Rcs system appears to be functional in the absence of RcsF, and it remains unknown how Rcs system senses signals that are independent of RcsF. In addition, similar to RcsC, RcsD also has a periplasmic domain. Whether the periplasmic domain of RcsD is also involved in sensing environmental signals and regulating Rcs system also needs to be clarified. Finally, many auxiliary proteins have now been identified, but how they are involved in the response to differential environmental signals and how they regulate physiological behavior in a coordinated manner to facilitate adaptation requires further investigation. In summary, the Rcs phosphorelay system provides a unique model for investigating the complexity of genetic regulation, environmental adaptation, and evolution in bacteria.

## Author Contributions

X-PG and Y-CS wrote the manuscript. Both authors have approved the final version for publication.

## Conflict of Interest Statement

The authors declare that the research was conducted in the absence of any commercial or financial relationships that could be construed as a potential conflict of interest.
